# Bioactive Compounds, Antibacterial, Antioxidant, Anticancer, and Antidiabetic Potential of the Seed and Leaves of *Tribulus terrestris*

**DOI:** 10.3390/life15121799

**Published:** 2025-11-24

**Authors:** Sahar Abdulaziz AlSedairy, Ibrahim M. Aziz, Rawan M. Alshalan, Mohamed A. Farrag, Abdulaziz M. Almuqrin, Amal Khalaf Alghamdi, Reem M. Aljowaie

**Affiliations:** 1 Department of Food Sciences and Nutrition, College of Food and Agricultural Sciences, King Saud University, Riyadh 11451, Saudi Arabia; 2 Department of Botany and Microbiology, College of Science, King Saud University, Riyadh 11451, Saudi Arabia; ralshalaan@ksu.edu.sa (R.M.A.); mfarrag@ksu.edu.sa (M.A.F.); raljowaie@ksu.edu.sa (R.M.A.); 3 Department of Clinical Laboratory Sciences, College of Applied Medical Sciences, King Saud University, Riyadh 12372, Saudi Arabia; aalmuqrin@ksu.edu.sa

**Keywords:** *Tribulus terrestris*, natural products, natural extracts, cancer, diabetes, apoptosis

## Abstract

Background: *Tribulus terrestris* is a medicinal plant used in traditional medicine to treat certain illnesses. Though past efforts mostly focused on the fruits and roots, current research examined the phytochemical composition and bioactivity of leaf extract (LE) and seed extract (SE). Methods: GC-MS compared phytochemical profiles, and total phenolic and flavonoid content were determined. The extracts were tested for antibacterial activity (disc diffusion, MIC/MBC), antioxidant potential (DPPH, ABTS^+^), cytotoxicity (MTT assay in MCF-7 and HepG2 cells), and anti-diabetic activity (α-amylase and α-glucosidase inhibition). Expression of apoptotic genes was also investigated. Results: The LE had a superior phytochemical composition, with greater phenolic and flavonoid levels. Compared to SE, it exhibited considerably higher antibacterial activity (MIC = 6.25–25 μg/mL), antioxidant potential (IC_50_ = 90.71–113.41 μg/mL), cytotoxicity (IC_50_ = 105.12–126.14 μg/mL), and enzyme inhibition (IC_50_ = 84–96.62 μg/mL). The LE also drastically reduced the expression of anti-apoptotic genes Bcl-2 and Bcl-xL in cancer cells. *T. terrestris* LE has significantly higher bioactive potential than SE in a range of pharmacological arenas due to its superior phytochemically complete profile. Conclusions: The findings indicate the LE as a promising candidate for the development of standardized phytotherapeutically active compounds.

## 1. Introduction

*Tribulus terrestris*, commonly known as *Gokshur* or *Gokharu* or puncture vine, is a herbaceous plant of the family *Zygophyllaceae* that is widely distributed in mild temperate and tropical regions, including parts of Europe, the United States, China, India, and the Mediterranean [1,2,3]. *T. terrestris* is utilized in traditional medicine as a tonic, aphrodisiac, palliative, astringent, stomachic, antihypertensive, diuretic, lithotriptic, and urinary disinfectant. The herb’s dried fruit is an effective treatment for most genitourinary tract disorders. It is a crucial part of Gokshuradi Guggul, a potent Ayurvedic treatment that promotes normal genitourinary tract function and gets rid of kidney stones. In Ayurveda, *T. terrestris* has been used for ages to cure sexual debility, venereal infections, and impotence. The herb is used as a traditional remedy for impotence in Bulgaria. The Indian Ayurvedic Pharmacopoeia assigns cardiotonic qualities to the fruit and root in addition to all these uses. The fruits were used in ancient Chinese medicine to treat morbid leukorrhea, sexual dysfunction, eye problems, edema, and abdominal distension. *T. terrestris* is recognized as a highly esteemed medicinal substance in the Shennong Pharmacopoeia, which is the earliest known pharmacological text in China, for its ability to rejuvenate the liver and treat conditions such as chest fullness, mastitis, flatulence, acute conjunctivitis, headaches, and vitiligo. In Unani medicine, *T. terrestris* is utilized as a diuretic, a mild laxative, and a general tonic [4,5,6].

Geographical regions play a crucial role in determining the composition of herbal pharmaceuticals. According to Dinchev et al. [7] (2008), proto-tribestin was found solely in samples sourced from Bulgaria, Turkey, Greece, Macedonia, Iran, and Serbia, with no traces of protodioscin in samples from Vietnam and India. This indicates that the compound could serve as a marker for the European strain of *T. terrestris* [8]. Lazarova et al. (2011) [9] revealed that there were significant variations across samples taken from the same country; for example, dioscin was not found in some samples taken from Bulgaria, and the chemicals’ amounts varied greatly. Because furostanol bidesmosides were converted into their spirostanol monodesmosides analogs during extraction, the outcome could be connected to the extraction techniques Lazarova and associates. Sarvin et al. (2018) demonstrated that a more extended extraction period (60 min) produced a greater yield, although Lazarova et al. (2011) [9], carried out the extraction by sonication for 15 min using 50% aqueous acetonitrile as a solvent [10]. Fruits, leaves, stems, and roots were found to contain the β-Carboline indole alkaloids harman, harmine, and harmalol; however, only the roots, stems, and leaves contained harmaline [5].

Due to its complex phytochemical profile, *T. terrestris* holds significant potential for medicinal applications. Steroid saponins and flavonoids are among the best-known and mostresearched of the plant’s many bioactive compounds [11]. The main active components of saponins are furostanol and spirostanol types, such as protodioscin and protogracillin. Their concentrations can vary significantly based on the plant’s geographic origin, the portion consumed, and the time of harvest [12]. The plant’s pharmacological qualities are also influenced by alkaloids such as harmane and norharmane, flavonoids derived from quercetin, kaempferol, and isorhamnetin, and other compounds like phytosterols and phenolic acids [13,14].

Recent scientific research has uncovered a wide range of pharmacological properties, supporting many of its traditional uses. Numerous studies have demonstrated the diuretic, anti-urolithic, anti-inflammatory, antioxidant, hepatoprotective, immunomodulatory, and antidiabetic qualities of *T. terrestris* extracts [1,11,15]. Although clinical evidence of its effectiveness in significantly raising human testosterone levels is still up for debate and needs more validation, its reputation for enhancing sexual function has been a significant focus of research, with studies examining its effects on erectile dysfunction and sperm parameters [16]. Furthermore, current research suggests its potential in other areas, such as cytotoxic activity against cancer cell lines, including liver cancer HepG2 cells, and wound-healing ability in diabetic models [17].

Even though traditional knowledge and growing phytopharmacological databases are available, there is a definite foundation for additional scientific research. There are relatively few thorough comparison studies examining distinctly different anatomical sections, such as the leaves and seeds, while the majority of published research has concentrated on the bioactivity of the roots and fruits. Furthermore, although a variety of biological activities have been identified, more research is needed to understand the mechanisms underlying these actions. Therefore, the goal of the current study was to compare the phytochemical and pharmacological profiles of the ethanolic extracts of *T. terrestris* leaf and seed, characterize their composition, and evaluate their cytotoxic, antioxidant, antibacterial, and antidiabetic properties.

## 2. Materials and Methods

### 2.1. Preparation of Ethanol SE and LE of T. terrestris

The leaves and seeds of *T. terrestris* were collected from El-Kharj region, Saudi Arabia. It was identified at the Botany and Microbiology Department’s Herbarium, King Saud University’s College of Science (KSU NO-147345). The leaves and seeds of Saudi *T. terrestris* were meticulously washed with distilled water, then air-dried at room temperature before being processed into a powder with an electric mixer. After that, the 20 g of plant powder was extracted using 100 mL of absolute ethanol. To remove contaminants and solid residues from the extract, Whatman No. 1 filter sheets were utilized. The extracts were then dried and concentrated using rotary vacuum evaporation (Yamato BO410, Yamato Scientific Co., Ltd., Tokyo, Japan). Finally, the dried extract was refrigerated at 4 °C for further use. The extract yield % was calculated using the following formula: Yield (%) = Weights of solvent-free extract (g) × 100/dried extract weight [18].

### 2.2. Determination of Bioactive Components

Bioactive components of *T. terrestris* LE and SE were identified by GC-MS (Santa Clara, CA, USA) using an Agilent Technologies system. To minimize the possibility of artifacts originating from plastic materials, all vials used were made of glass, and procedural blanks were run under identical conditions to ensure that no contamination peaks overlapped with those of the plant extracts. Briefly, 2 mg of each dried extract was dissolved in 2 mL of high-performance liquid chromatography (HPLC)-grade methanol, filtered through a 0.22 µm PTFE membrane filter, and 1.5 µL of this filtrate was injected via an autosampler injection system of GC-MS 7890B GC system from Agilent Technologies (Santa Clara, CA, USA). The products were identified using the database-integrated software (NIST MS). The identification of the sample components was achieved using Gas Chromatography coupled with a mass selective detector (GC-MS). For the separation of target compounds, a DB-5 MS fused silica capillary column (30 m × 0.25 mm, 0.25 μm) was used, with helium as the carrier gas at 1 mL/min (for 1 min). The oven temperature program began with 3 min at 40 °C, increasing by 7.5 °C per minute to 280 °C (held for 5 min), then to 290 °C (held for 1 min). The injector and detector temperatures were set to 200 °C and 300 °C. Data were collected in electron impact (EI) mode at 70 eV, scanning *m*/*z* 91–283. The split injection ratio was 1:10 (1 μL volume), and the total run time was 60 min. The MS detector was set as follows: Acquisition scan type, mass ranging from 40 to 500 g/mol, scan speed 1.56, 8 min solvent delay, and 230 °C MS source temperature. Compounds were identified by comparing spectra with Wiley and NIST mass libraries, considering matches above 90% determined.

### 2.3. Determination of Total Phenolic Content (TPC) and Total Flavonoid Content (TFC)

The TPC values of *T. terrestris* LE and SE were determined using the Folin–Ciocalteu standard technique [19] with slight modifications. In brief, a solution of 0.1 mL of plant extract (1 mg/mL) and 3 mL of distilled water was mixed. Five minutes later, 2 mL of 20% (*w*/*v*) sodium carbonate (Na_2_CO_3_) was added. At 25 °C, the reaction mixture was incubated in the dark for 30 min. Utilizing a spectrophotometer (U2001 U2001 UV-VIS-Spectrophotometer, Hitachi, Tokyo, Japan), the absorbance at 725 nm was measured. Gallic acid was used as a reference, and the results are presented as milligrams of gallic acid equivalents per gram of dry weight (mg GAE/g DW).

The aluminum chloride (AlCl_3_) colorimetric assay, as described in reference [20], was used for TFC measurement with slight modifications. Briefly, 1 mL of 2% AlCl_3_ was mixed with 500 μL of the leaf and seed extracts (2 mg/mL). The mixture was then mixed with 3 mL of sodium acetate solution (50 g/L). Following that, two acetic acid drops were added. Following a one-hour dark incubation period at 25 °C, the mixture’s absorbance was measured at 420 nm using a spectrophotometer (U2001 UV-VIS-Spectrophotometer, Hitachi, Japan). Quercetin was chosen as the standard, and the TFC was expressed as quercetin equivalents in milligrams per gram of dry sample (mg QE/g DW).

### 2.4. Antibacterial Activity

#### 2.4.1. Disc Diffusion Method

The antibacterial activity of the LE and SE of *T. terrestris* was assessed by disc diffusion assay as previously described [21], against 3 Gram-positive bacteria: *Staphylococcus aureus* (MTCC-29213), *Staphylococcus epidermidis* (MTCC-12228), *Bacillus subtilis*, (MTCC-10400) and 3 Gram-negative bacteria; *Escherichia coli* (ATCC-25922), *P. aeruginosa,* (MTCC-27853), *Klebsiella pneumonia* (MTCC-13883). Muller-Hinton broth (MHB) was used to cultivate the examined bacteria, which were then incubated for 24 h at 37 °C. Mueller-Hinton agar (MHA) was then stirred with a 0.1 mL bacterial solution (at a McFarland turbidity of 0.5). A sterile corkborer was used to punch 5 mm-diameter holes at equal intervals to form wells. This was followed by adding various concentrations of *T. terrestris* LE and SE (100, 200, 400, and 800 µg/mL) to the wells. Chloramphenicol (25 µg/mL) served as the positive control, while Muller-Hinton Broth (HMB) served as the negative control. All of the plates were incubated at 4 °C for two hours, then incubated at 37 °C for twenty-four hours to promote microbial growth and estimate the zone of inhibition surrounding each well. The millimetres (mm) of the inhibitory zone that formed around the discs were measured. Three separate tests were conducted.

#### 2.4.2. Determination of Minimum Inhibitory Concentration (MIC) and Minimum Bactericidal Concentration (MBC)Values

The MIC of the LE and SE were determined against the previously mentioned bacterial strains using the microdilution broth technique in a 96-well microplate. The assay used 2,3,5-triphenyltetrazolium chloride (TTC) as described by Basri and Sandra (2016), with minor modifications [22]. Two hundred microliters of MHB medium were added to each well of the microplate. The LE and SE were tested using a two-fold serial dilution (1.95–1000 µg/mL). Chloramphenicol (25 µg/mL) served as the positive control for the MIC assay. After adjusting the bacterial cell solution to 10^6^ CFU/mL, 10 µL was added to each well. Microplates were incubated at 37 °C for 24 h. Then, 20 µL of TTC (2 mg/mL) was added to each well. The appearance of a red color indicated bacterial proliferation. The lowest concentration with no observable color change was recorded as the MIC. To determine the MBC, 100 µL from wells without color change were cultured on MHA and incubated at 37 °C for another 24 h [23].

### 2.5. Antioxidant Activity

#### 2.5.1. Diphenyl-1-Picrylhydrazyl (DPPH) Radical Scavenging Activity

The ABTS^+^ cation radical decolorization technique was used to assess the antioxidant efficacy of *T. terrestris* LE and SE. This approach was carried out in accordance with the protocol outlined in the earlier study [24]. Various concentrations of *T. terrestris* LE and SE were prepared (100, 200, 400, and 800 µg/mL). To each concentration of the extract, 2 mL of the 0.08 mM DPPH solution was added, and the mixture was vigorously agitated. Ascorbic acid (200 μL, 100–800 μg/mL) was used as the standard control. The reaction mixtures were allowed to rest for 30 min at 25 °C. After the incubation period, the optical density (OD) of the samples was measured at 517 nm using a U2001 UV-VIS (U2001) Hitachi, Japan). The IC_50_ values for ascorbic acid and *Moringa oleifera* extract, indicating the concentration required to reduce the initial DPPH concentration by 50%, were determined using Graph Pad Prism software (version 5.0, La Jolla, CA, USA). The antioxidant activity of the *T. terrestris* LE and SE was evaluated using the following formula: DPPH radical scavenging activity (%) = (OD of control − absorbance of the extract)/OD of control × 100.

#### 2.5.2. 2,2′-Azino-bis(3-ethylbenzothiazoline-6-sulfonic acid) (ABTS) Assay

To evaluate the antioxidant activity of *T. terrestris* LE and SE, the ABTS^+^ cation radical decolorization assay was used. This method was performed according to the procedure described in a previous study [25]. The concentrations of ascorbic acid and *T. terrestris* LE and SE were established as follows: 100, 200, 400, and 800 µg/mL. The ABTS solution (192 mg in 50 mL of distilled water) was mixed with 140 mM. The ABTS solution (192 mg in 50 mL of distilled water) was mixed with the 140 mM K_2_S_2_O_8_ solution (K_2_S_2_O_8_) in the dark for 12–16 h at 25 °C to generate the ABTS cation radical (ABTS^+^). This radical solution was subsequently diluted in ethanol (1:89, *V*/*V*) to obtain an OD of approximately 0.70  ±  0.02 at 734 nm. The assay involved the mixing of 1 mL of the diluted ABTS^+^ solution with 1 mL of each concentration of *T. terrestris* LE and SE, or ascorbic acid. A spectrophotometer was employed to measure the OD at 734 nm after the reaction mixtures were allowed to equilibrate at 30 °C. The readouts of ABTS^+^ % and IC_50_ were presented as described above.

### 2.6. Cell Culture and Cytotoxicity Assays

The cytotoxic properties of extracts derived from the LE and SE of *T. terrestris* were evaluated in vitro utilizing human hepatoma HepG2 (ATCC HB-8065) and breast cancer MCF-7 (ATCC HTB-22) cellular models [26,27]. The cells are maintained at 37 °C in 5% CO_2_ in Dulbecco’s modified Eagle medium (DMEM) supplemented with 1% penicillin-streptomycin and fetal calf serum (FCS). The 3-(4,5-dimethylthiazol-2-yl)-2,5-diphenyl-2H-tetrazolium bromide (MTT) assay was used to assess the cell viability [28]. Various concentrations of *T. terrestris* LE and SE (125, 250, 500, and 1000 µg/mL) were added to the culture medium, and the cells were incubated for 24 h at 37 °C with 5% CO_2_. The positive control was cisplatin (30 µg/mL). The cells were not exposed to *T. terrestris* LE and SE, which acted as negative control cells. After incubation, 10 µL of the MTT solution (5 mg/mL) was applied to each well. Each well received 10 µL of the MTT solution (5 mg/mL) following incubation. The cell culture plates were subjected to an operational shaker (MPS-1, Biosan, London, UK) at 150 rpm for 5 min to mix the components effectively, followed by an incubation period of 2–4 h. The MTT solution was then removed, and 100 µL of Dimethyl sulfoxide (DMSO) was added to each well. The optical density (OD) for each treatment was measured at 570 nm using an ELX-808 microplate reader (BioTek Laboratories, LLC., Shoreline, WA, USA), with a reference wavelength of 620 nm. The percentage of cell viability and cell death was calculated using the following formulas:

The cell viability (%) = [(OD of treated cells − absorbance of the extract)/OD of Untreated cells (control)] × 100. GraphPad Prism software (version 5.0, La Jolla, CA, USA) was used to calculate the IC_50_ value, with the mean value ± SD for data processing [29].

### 2.7. Antidiabetic Activity

#### 2.7.1. Determination of α-Amylase Inhibitory Activity

The inhibitory activity of α-amylase by extracts from LE and SE of *T. terrestris* was assessed utilizing the 3,5-dinitrosalicylic acid (DNSA) method [30]. In summary, the plant extracts were diluted to achieve concentrations between 50 and 1000 μg/mL with a buffer solution (0.02 M Na_2_HPO_4_/NaH_2_PO_4_; 0.006 M NaCl; pH 6.9). The mixture was incubated for 10 min at 37 °C after combining 200 µL of each extract with 200 µL of the Molychem α-amylase solution (2 units/mL). Subsequently, each tube was filled with 200 µL of a 1% starch solution (*w*/*v*) and incubated at 37 °C for 3 min. To halt the reaction, 200 µL of DNSA reagent (composed of 12 g of sodium potassium tartrate tetrahydrate in 8.0 mL of 2 M NaOH and 20 mL of 96 mM 3,5-DNSA solution) was added, followed by heating for 10 min at 85 °C in a water bath. The positive control consisted of 100 μL of 400 µg/mL acarbose (Bayer). After allowing the sample to cool to room temperature and diluting it with 5 mL of distilled water, the optical density at 540 nm was recorded using aU2001 UV-VIS-Spectrophotometer (U2001 UV-VIS-Spectrophotometer, Hitachi, Japan). The following formula computed the inhibition of α-amylase as a percentage.

The inhibitory activity of extract (%) = [(X − Y)/X] × 100, where X denotes the reaction occurring without the extract, while Y indicates the enhancement in absorbance when the extract is present.

The IC_50_ values were determined using GraphPad Prism software (version 5.0, La Jolla, CA, USA).

#### 2.7.2. Determination of α-Glucosidase Inhibitory Activity

The α-glucosidase inhibitory activity of *T. terrestris* LE and SE was measured using yeast α-glucosidase and p-nitrophenyl-α-D-glucopyranoside (pNPG) as previously reported [31]. To get 0.5 to 5.0 mg/mL final concentration, 50 μL of α-glucosidase (1 U/mL) produced in 0.1 M phosphate buffer (pH 6.9) and 250 μL of 0.1 M phosphate buffer were added to the ethanol flower extract of *M. recutita* L. or Acarbose (a positive control) (100 μL of 2 to 20 mg/mL). The mixture was pre-incubated for twenty minutes at 37 °C. Ten μL of 10 mM pNPG made in 0.1 M phosphate buffer (pH 6.9) were added after pre-incubation and left for 30 min at 37 °C. The reactions were terminated by adding 650 μL of 1 M sodium carbonate, and the absorbance was measured at 405 nm using A UV-vis spectrophotometer (U2001 UV-vis Spectrophotometer, Hitachi, Japan). The percentage of inhibition of enzyme activity and the IC_50_ values were calculated as described above.

### 2.8. Statistical Analysis

GraphPad Prism (version 5.0, La Jolla, CA, USA) was used to analyze the results. The mean value ± standard deviation (SD) of three independent experiments in triplicate was presented. Student’s unpaired *t*-test was used to compare the means of two independent groups. For non-Gaussian variables, the Mann–Whitney U test was used to compare the groups. Significant differences between the means of the measurements were determined using an LSD test, with *p* ≤ 0.05 considered statistically significant. All measurements were performed in triplicate.

## 3. Results

### 3.1. Extraction Yields

The yield of the *T. terrestris* LE and SE was 12.13% and 12.47%, respectively, based on the applied operating mode and the dry matter weight computation (*w*/*w*).

### 3.2. Chemical Composition of the T. terrestris LE and SE

**Figure 1:**
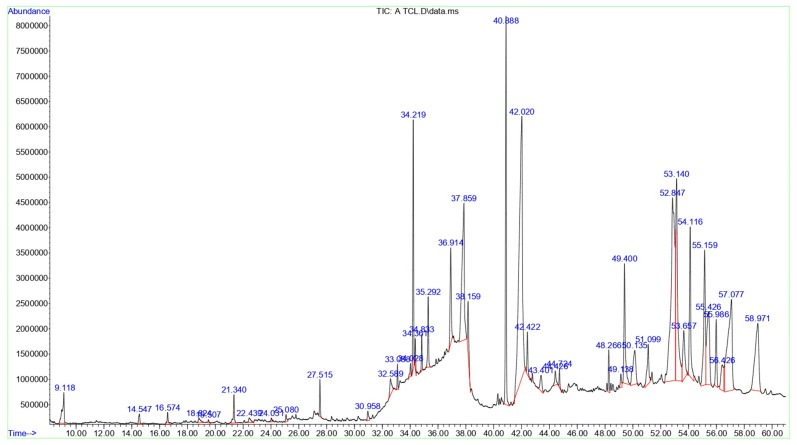
The GC-MS chromatograms of *T. terrestris* LE. All spectral peaks correlate with the identified chemicals, with a major peak indicating the primary constituent of the extract.

GC-MS analyses were performed in triplicate for both extracts, yielding extremely consistent chromatographic profiles with relative peak-area standard deviations < 5%. There were no contamination peaks in solvent blanks, indicating that the detected components came from plant sources. The bioactive compounds in *T. terrestris* LE were identified using GC–MS. The GC–MS chromatogram depicting these bioactive compounds is displayed in Figure 1, with their peak retention time (RT), peak area (%), molecular formula (MF), and molecular weight (MW) listed in Table 1. 42 bioactive compounds were identified in *T. terrestris* LE, expressed as percentages of the peak area relative to the overall peak area. The predominant constituent in the *T. terrestris* leaves was Linolenic acid, which accounted for 16.32% of the total. The following constituents were Vitamin E (13.49%), Campesterol (7.46%), Linolenic acid, methyl ester (6.99%), Phytol (6.47%), and n-Hexadecanoic acid (6.09%). Collectively, the main categories in the GC-MS analysis of *T. terrestris* LE were Lipids and Fatty Acid Derivatives (37.08%), followed by Terpenoids and Steroids (26.48%) and Glycosyl Compounds and Carbohydrates (8.31%).

**Figure 2:**
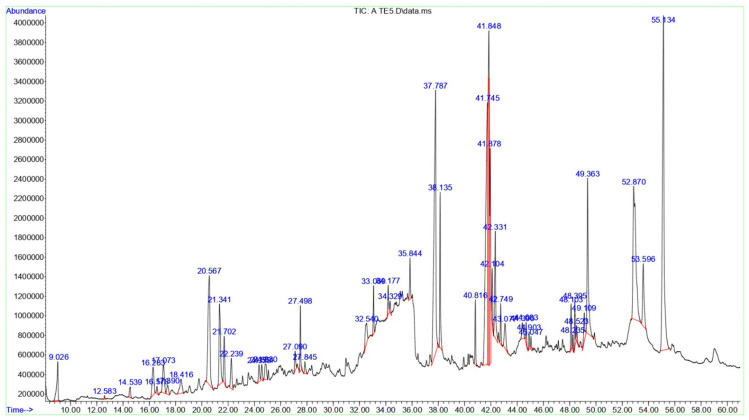
The GC-MS chromatograms of *T. terrestris* SE. A prominent peak in the spectrum indicates the major component of the extract, and every peak reflects a recognized chemical.

However, a total of 46 bioactive components have been discovered in the *T. terrestris* SE, with Oleamide being the predominant compound, representing 13.01% of the total. The following compounds were cis,cis-Linoleic acid (12.05%), n-Hexadecanoic acid (11.38%), and 1-Monolinolein (8.68%), Linolenic acid (7.69%), and Linoleic acid ethyl ester (5.33%) (Table 2 and Figure 2). Collectively, the main categories in the GC-MS analysis of *T. terrestris* SE were the Lipids and Fatty Acid Derivatives composed the highest percentages (55.71%), followed by Glycerolipids (16.54%), and Glycosyl Compounds and Carbohydrates (3.08%), where Terpenoids and Steroids were represented by only 1.89%.

### 3.3. TPC and TFC of T. terrestris LE and SE

The TPC and TFC assay results revealed a significant difference in phytochemical content between the *T. terrestris* LE and SE. The TPC for the *T. terrestris* LE was higher than that of the *T. terrestris* SE (68.12 mg GAE/g DW). Similarly, the TFC for the *T. terrestris* LE was found to be (87 mg QE/g DW) higher than that of the *T. terrestris* SE (61 mg QE/g DW).

### 3.4. Antibacterial Effects of T. terrestris LE and SE

The disc diffusion technique was used to assess the antibacterial properties of the ethanol extracts of *T. terrestris* LE and SE against various bacterial strains. Table 3 and Table 4 demonstrate this extract’s ability to prevent the growth of the tested bacteria. The findings demonstrated that the extracts inhibited the growth of bacterial strains in a dose-dependent manner at various concentrations. The antibacterial activity of *T. terrestris* LE and SE increased gradually with higher concentration; at 400 and 800 μg/mL, the inhibitory zones began to increase significantly (*p* < 0.05), although not as much as the positive control (25 µg/mL of chloramphenicol). The *T. terrestris* LE showed more antibacterial activity with MIC 6.25 ± 0.00–25 ± 0.00 μg/mL than *T. terrestris* SE with MIC = 12.50 ± 0.00–50 ± 0.00 μg/mL. Gram-negative bacteria, particularly *K. pneumoniae* were more vulnerable to *T. terrestris* LE or SE.

### 3.5. DPPH and ABTS+ Radical Scavenging Activity

**Figure 3:**
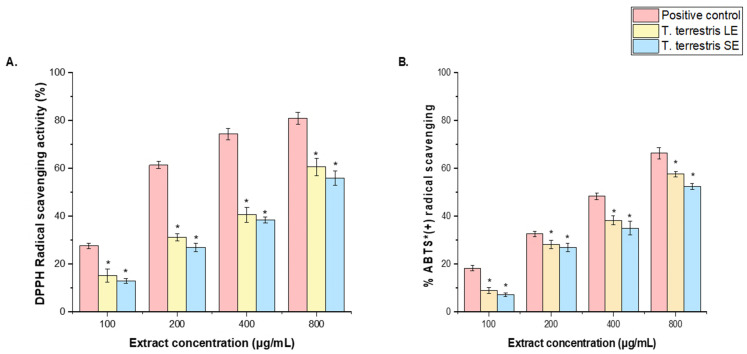
Antioxidant activity of *T. terrestris* LE and SE. (**A**) DPPH reducing power and (**B**) ABTS^+^ scavenging activity at various concentrations (100–800 μg/mL). An ascorbic acid (100–800 µg/mL) was used as a positive control. The mean value of 3 independent experiments is presented. The scavenging activity of *T. terrestris* LE and SE was significantly lower (*) than the positive control at a significance level of *p* < 0.05. + = radical cation.

DPPH and ABTS^+^ assays were performed to evaluate the radical-scavenging properties of the *T. terrestris* LE and SE, expressed as IC_50_ values, and compared with ascorbic acid, the representative positive control for these assays. Based on the IC_50_ values in the DPPH and ABTS^+^ assays. The *T. terrestris* LE with IC_50_ value (113.41 μg/mL) demonstrated higher antioxidant ability than the *T. terrestris* SE IC_50_ = 162.42 μg/mL). However, the IC_50_ value of both the T. *terrestris* LE and SE is lower than the antioxidant level of the positive control (IC_50_ = 28.11 μg/mL) (Figure 3A,B).

Likewise, the IC_50_ value for the *T. terrestris* LE (90.71 μg/mL) exhibited superior antioxidant activity compared to the IC_50_ value of the T. terrestris SE (176.11 μg/mL) in the ABTS + radical assay. However, both the *T. terrestris* LE and SE have IC_50_ values that are lower than the antioxidant level of the positive control (IC_50_ = 24.18 μg/mL) (Figure 3A,B). The observed variation in DPPH and ABTS^+^ radical-scavenging activity between the LE and SE of *T. terrestris* may be due to differences in the quantity of phytochemicals in each extract.

### 3.6. Cell Cytotoxicity

**Figure 4:**
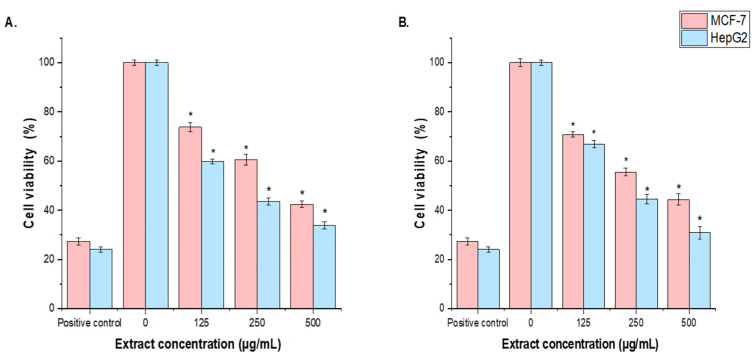
The effect of *T. terrestris* (**A**), LE and (**B**), SE on MCF-7 and HepG2 cell viabilities using the MTT assay. Cells were treated with the *T. terrestris* LE and SE (125, 250, 500, and1000 µg/mL) for 24 h. Mean values ± SD of three independent experiments are shown. (* *p* < 0.05 compared to non-treated cells (negative control)).

The cytotoxicity activity of *T. terrestris* LE and SE against MCF-7 and HepG2 cells was assessed using the MTT assay. *T. terrestris* LE and SE were tested for their anti-carcinogenic properties at concentrations of 125, 250, 500, and 1000 µg/mL. The cytotoxic activity of *T. terrestris* leaf and seed extracts on MCF-7 and HepG2 cells was concentration-dependent. At 125 μg/mL of *T. terrestris* LE and SE, cellular proliferation of MCF-7 and HepG2 lines was significantly (*p* < 0.05) inhibited compared to the untreated control cells, although less than the positive control (30 µg/mL of cisplatin). The cytotoxic effects of the *T. terrestris* LE were more potent than those of the *T. terrestris* SE, with IC50 values of 126.14 μg/mL and 105.12 μg/mL against MCF-7 and HepG2 cells, respectively, in both tests. Interestingly, the current investigation showed that HepG2 cells were more responsive to LE and SE than MCF-7 cells (Figure 4A,B).

**Figure 5:**
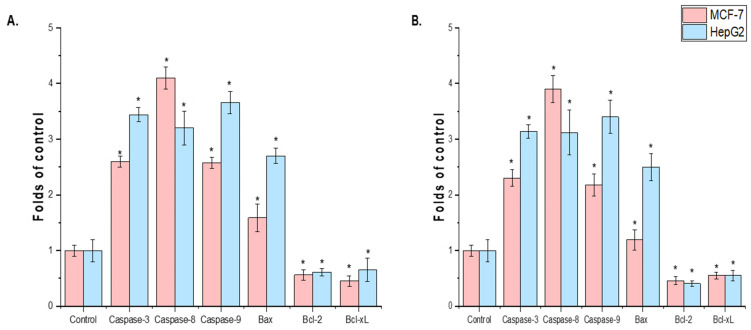
Effect of *T. terrestris* (**A**), LE and (**B**), SE on MCF-7 and HepG2 cells and determination of pro- and anti-apoptosis marker genes (caspase-3, 8, and 9, Bax, Bcl-2, and *Bcl-Xl* genes). The values represent the mean ± SD from ± trials (* *p* < 0.01).

Ethanol extracts from *T. terrestris* seeds and leaves were evaluated for their effects on HepG2-induced apoptotic signaling and MCF-7. The MCF-7 and HepG2 cell lines treated with seed extract showed higher levels of mRNA expression, according to the rRT-PCR study. Anti-apoptotic genes (Bcl-xL and Bcl-2) were expressed less in the MCF-7 and HepG2 cell lines treated with plant extract than in the control group (*p* < 0.05) (Figure 5A,B).

### 3.7. In Vitro Antidiabetic Activities of T. terrestris LE and SE

**Figure 6:**
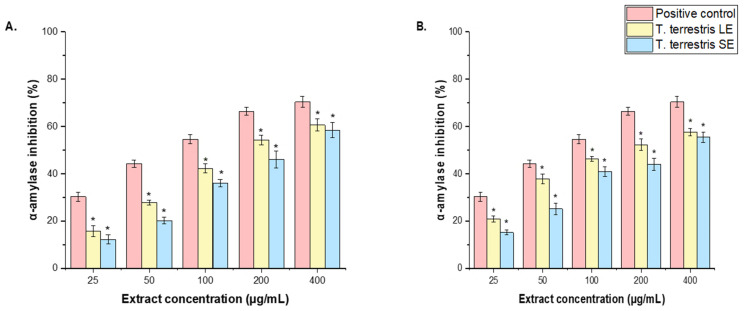
Effect of *T. terrestris* (**A**), LE and (**B**), SEon α-amylase and α-glucosidase inhibitory activities at various concentrations (25–400 μg/mL). The results are the mean values of three replicates. The results are the mean ± SD of three experiments (* *p* < 0.05 compared to the acarbose, positive control).

The antidiabetic activities of *T. terrestris* LE and SE were determined using the inhibitory effects on *α*-amylase and α-glucosidase enzymatic activities. The results are expressed as IC_50_ values. The *T. terrestris* LE showed a considerable inhibitory effect against α-amylase and α-glucosidase (IC_50_ = 84 ± 2.84 μg/mL and 96.62 ± 1.13 μg/mL, respectively) (Figure 6A), whereas *T. terrestris* SE inhibited α-amylase (IC_50_ = 113 ± 0.14 μg/mL), α-glucosidase (IC_50_ = 126.62 ± 1.12 μg/mL) (Figure 6B). The LE showed a significant inhibitory action against both enzymes (IC_50_ = 84 ± 2.84 μg/mL and 96.62 ± 1.13 μg/mL, respectively) in comparison to the *T. terrestris* SE, which had IC_50_ values of 113 ± 0.14 μg/mL for α-amylase and 126.62 ± 1.12 μg/mL for α-glucosidase. Conversely, both the LE and SE of *T. terrestris* presented IC_50_ values for the two enzymes analyzed, α-amylase (IC_50_ = 34.41 ± 0.18 μg/mL) and α-glucosidase (IC_50_ = 42.52 ± 1.28 μg/mL), that were less than the positive control, which was noted at 28.11 μg/mL (as shown in Figure 6A,B).

## 4. Discussion

The GC-MS analysis of *T. terrestris* LE and SE revealed a complex phytochemical profile dominated by specific chemical classes, many of which are known to have biologically relevant properties. In the current study, Linolenic acid was represented in the LE and SE by 16.32% and 12.05%, respectively. In the LE, Vitamin E, Campesterol, and Phytol were the most represented compounds by 13.49%, 7.46%, and 6.47%, respectively. The highest expression levels in the SE were 13.01%, 11.38%, and 8.68% for oleamide, n-hexadecanoic acid (palmitic acid), and 1-monolinolein, respectively. The potential for antibacterial properties of the LE and SE is greatly enhanced by the high concentration of fatty acids in both [32,33]. Fatty acid components in a recent study on the fruit extract of *T. terrestris* demonstrated possible antibacterial activity against methicillin-resistant *S. epidermidis* (MRSE) according to HPLC fractionation [34]. The presence of Vitamin E and terpenoids such as Phytol in the LE is consistent with the potent antioxidant activity reported for several *T. terrestris* extracts. Studies have indicated that ethanol and methanol extracts of the plant showed strong radical scavenging capacities in DPPH, FRAP, and ABTS assays, which are crucial for alleviating oxidative stress [35,36,37]. The presence of Oleamide in the SE and other fatty acids in both extracts is consistent with previous findings about their effect on the inflammatory pathways [36]. The high concentration of sterols in LE, such as Campesterol and Stigmasterol, is notable. These substances are structurally similar to the steroidal saponins (e.g., dioscin), which have been identified as the principal anticancer agents in *T. terrestris*, triggering apoptosis in cancer cells [5]. n-Hexadecanoic acid and monoacylglycerols, such as 1-Monolinolein, have been shown to inhibit enzymes, including α-glucosidase [37]. *T. terrestris* extracts, particularly ethanol extracts, have been shown to have a substantial inhibitory effect against α-glycosidase and cholinesterases (AChE and BChE), which are essential for managing diabetes and Alzheimer’s disease, respectively [37]. An in vivo investigation verified the antihyperglycemic efficacy of an aqueous extract of *T. terrestris*, which dramatically reduced blood glucose and glycated hemoglobin (HbA1c) levels in diabetic rats [38].

The phytochemical profile is consistent with previous investigations on *T. terrestris*, while exhibiting noticeable variations. According to a detailed examination, the chemical makeup of the plant varies greatly depending on the plant component, geographic origin, and extraction solvent. For example, although the LE has Vitamin E and β-Lactose, fruit extracts from other locations typically contain steroidal saponins such as protodioscin [5]. These findings, indicating a significant concentration of fatty acids and lipids in the seeds, are consistent with the plant’s biology, as seeds commonly store energy as lipids [5]. Ethyl iso-allocholate and digitoxin were found to be more prevalent in the GC-MS analysis of alcoholic extracts of *T. terrestris* leaves and fruits, according to another study [39]. *T. terrestris’* medicinal potential reflects the *Zygophyllaceae* family’s overall pharmacological significance. The presence of potent secondary metabolites, such as saponins, flavonoids, and alkaloids, is a common feature across many genera in this family, supporting their use in traditional medicine systems worldwide [40]. The GC-MS profiles provide a preliminary screening of *T. terrestris*’ volatile and semi-volatile metabolites. While some fatty acids, sterols, and terpenoids were clearly detected, which is compatible with other previous studies [41,42], the presence of certain chemicals (e.g., oleamide or arachidonic acid derivatives) should be regarded with caution, as they could be the result of minor analytical errors or derivatization reactions during the GC-MS procedure. However, careful use of glassware and procedural blanks reduced the risk of contamination as mentioned in the Section 2 .

In the current study, both extracts showed higher TPC and TFC values, but these were higher in the LE compared to the SE. That suggests a phytochemical basis for *T. terrestris*’ powerful biological activities. The flavonoids in *T. terrestris* are mainly derivatives of quercetin, kaempferol, and isorhamnetin [4]. A previous study showed that a difference in the polarity of the extraction solvents might affect the amounts of flavonoids and phenols measured, where the ethanolic extract resulted in higher values (TPC: 51 ± 0.7 mg. GAE/g and TFC: 66.5 ± 0.4 mg QE/g) compared to aceton (TPC: 47 ± 1.5 mg. GA. E/g and TFC: 52.5 ± 0.5 mg QE/g) and chloroform (TPC: 37 ± 1.2 mg. GA. E/g and TFC: 43 ± 1.5 mg QE/g) extracts [43]. According to another study, the TPC of various *T. terrestris* LE varied between 17.93 and 32.00 mg GAE/g, with the methanolic extract having the highest TFC at 27.62 mgRE/g [44]. So, TFC levels in the LE not only confirm a higher concentration of polyphenolic antioxidants, but also clearly suggest that the leaves are a more attractive source for leveraging the plant’s established anti-inflammatory and anticancer effects than the SE.

The preliminary phytochemical research on *T. terrestris* revealed the presence of saponins, flavonoids, glycosides, alkaloids, and tannins [45]. Data from the literature indicate that *T. terrestris* from various geographical locations has varying saponin contents and compositions [7]. The chemistry and bioactivity of saponins in *T. terrestris* were investigated by Kostova et al. According to their findings, this plant commonly contains furostanol and spirostanol saponins of the tigogenin, neotigogenin, gitogenin, neogitogenin, hecogenin, neohecogenin, diosgenin, chlorogenin, ruscogenin, and sarsasapogenin kinds. Four sulfated saponins of the diosgenin and tigogenin types were also identified. Furostanol glycosides, such as protodioscin and protogracillin, are mostly found; protodioscin is the most prevalent saponin. Spirostanol glycosides are found in trace amounts [46,47]. According to Wu et al., there are almost 1.5 times as many major flavonoids as main saponins. This suggested that the flavonoid content of *T. terrestris* should be investigated, developed, and further utilized [48]. In leaf extracts from four *Tribulus* species, Louveaux et al. used HPLC to identify eighteen flavonoids (caffeoyl derivatives, quercetin glycosides, including rutin and kaempferol glycosides) [49].

The antioxidant activity of *T. terrestris* LE and SE differs significantly. The LE (IC_50_ = 113.41 μg/mL in DPPH; 90.71 μg/mL in ABTS^+^) outperformed the SE (IC_50_ = 162.42 μg/mL in DPPH; 176.11 μg/mL in ABTS^+^). The DPPH assay assesses hydrogen-atom transfer activity, while the ABTS^+^ assay detects electron-transfer capacity [50], resulting in different sensitivity profiles for the same complex mixture. This found bioactivity is consistent with the plant’s known phytochemistry and the *Zygophyllaceae* family’s overall pharmacological profile. Previous studies showed that *T. terrestris’* primary antioxidants are steroidal saponins (e.g., protodioscin) and flavonoids (e.g., rutin, quercetin, isorhamnetin) [4,36]. That a methanolic extract of *T. terrestris* had significant phenolic (341.3 mg GAE/g) and flavonoid (209 mg QE/g) content, which correlates with strong antioxidant activity. The LE is more potent in the current study, showing that it contains a larger concentration of these phenolic and flavonoid chemicals than the SE. From another point of view, saponin-rich *T. terrestris* fractions showed higher antioxidant and anti-glycation activity than crude extracts [51,52]. They showed that increasing the saponin content from 40% to 72.8% resulted in an 89.89% reduction in advanced glycation end-product and improved performance in several antioxidant tests. Furthermore, while there have been few systematic comparative studies about the antioxidant activities of *Zygophyllaceae* members, this family is known for producing a wide range of bioactive chemicals of known antioxidant potential. Different extracts from *Zygophyllum Geslini* Coss [53], *Zygophyllum simplex* L. [54], and *Zygophyllum coccineum* L. [55], showed significant antioxidant activities due to their robust chemical profile. The antioxidant potential of these extracts might be explained through various routes, including direct free radical scavenging and enhancing the body’s endogenous defence by increasing glutathione levels. In the study conducted by Shetty et al. (2024) [56], they found that *T. terrestris*’ nephroprotective and hepatoprotective benefits in rats were achieved by regulating the inflammatory marker IL-6 and lowering oxidative stress. The LE’s enhanced antioxidant activity is most likely owing to a greater concentration of steroidal saponins and flavonoids [56].

The current study evaluated the antibacterial properties of *T. terrestris* LE and SE. The results showed that the LE was more potent than that of the SE with MICs of 6.25–25 μg/mL and 12.50–50 μg/mL, respectively. The most susceptible strains were *E. coli* for the seed extract and *K. pneumoniae* for both. On the other side, *S. aureus* and *S. epidermidis* were the most resistant to the LE, while *B. subtilis* was the most resistant to the SE. The findings are consistent with the previously documented antibacterial profile of *T. terrestris*. Al-Bayati and Al-Mola (2008) [32], conducted a study in Iraq. They found that, to the root extract, the methanolic leaf had the lowest MIC values against *B. subtilis* (0.31 mg/mL) and *K. pneumoniae* (0.31 mg/mL). According to a different study, the antibacterial activity of *T. terrestris* fruit extract against *Streptococcus mutans*, *Streptococcus sanguis*, *Actinomyces viscosus*, *Enterococcus faecalis*, *S. aureus*, and *Escherichia coli* was enhanced when combined with *Glycyrrhiza glabra* and *Capsella bursa-pastoris* [33]. A recent study found that the methanolic extract of *T. terrestris* fruit significantly inhibited the acidification activity of *S. mutans* and *Lactobacillus acidophilus*, two oral microbes that cause cavities in teeth (Azarm et al., 2024) [57]. It is believed that *T. terrestris’s* potent antibacterial properties stem from its diverse phytochemical makeup. Numerous bioactive compounds, such as alkaloids, flavonoids, and saponins, are said to be present in the plant. The compounds from a fruit fraction were effective against MRSE in the study by Zhu et al. (2017) [4]. By targeting the penicillin-binding protein 2a (PBP2a) transpeptidase, a critical protein mediating methicillin resistance, these bioactive compounds may inhibit bacterial growth, according to a recent study that used molecular docking tools [34]. So, these findings provide strong experimental evidence to support the traditional use of *T. terrestris* and highlight its leaves as a ting source of antibacterial agents with various modes of action.

The results demonstrated that *T. terrestris* LE and SE exhibit concentration-dependent cytotoxicity against MCF-7 and HepG2 cell lines. A similar study found substantial benefits across multiple cancer types. A methanolic extract of *T. terrestris* exhibited cytotoxicity against lung cancer A549 cells (IC_50_ = 179.62 μg/mL) and MCF-7 cells [58]. Moreover, exceptional efficacy has been found against additional lines, such as human ovarian adenocarcinoma A2780 cells (IC_50_ = 3.69 μg/mL) [17], colorectal cancer HT-29 cells (IC_50_ = 7.1 μg/mL), and prostate cancer LNCaP cells (IC_50_ = 0.3 μg/mL) [59]. The gene expression data revealed downregulation of the anti-apoptotic genes Bcl-2 and Bcl-xL, which is central to the mechanism of action. Previous studies found that *T. terrestris* extracts greatly increased the activity of important executioner enzymes, caspase-3 and caspase-8 [58,60], and upregulate key pro-apoptotic proteins such as Bax and p53 [60], further shifting the cellular balance toward programmed cell death. The mitochondrial (intrinsic) apoptotic pathway in cancer cells is successfully started by this dual action of blocking survival signals (Bcl-2/Bcl-xL) and activating cell death executors (caspases, Bax). Multiple studies have shown that *T. terrestris* extracts have much lower cytotoxicity against non-malignant cells. This includes PBMCs and normal fibroblast cells (L929 and HSkMC) [58,60], which were significantly more viable than cancer cells after treatment. However, a word of caution is required; one study on human cells found that high amounts of aqueous fruit extract (40–80 mg/L) may have genotoxic effects, such as producing micronuclei and chromosomal abnormalities [61,62] demonstrated that *Tribulus macropterus*, a close relative of *T. terrestris*, exhibited similar cytotoxic activity against HepG2 liver cancer cells, attributed to the presence of cytotoxic cholestane and pregnane glycosides. *Nyctanthes arbor-tristis* (Oleaceae) also exhibits alkaloid extracts that induce apoptosis by changing the Bax/Bcl-2 ratio [63]. This shows that many therapeutic plants use the same successful approach to target the apoptotic machinery.

*T. terrestris* LE and SE have been shown to inhibit α-amylase and α-glucosidase, providing quantitative evidence for their traditional usage in diabetes management. The observed differences in efficacy between plant sections, in contrast to a typical medicine, provide valuable information for medicinal development. A previous study discovered that a methanolic root extract of *T. terrestris* significantly inhibited α-glucosidase [17]. Another study showed that ethanol extracts of *T. terrestris* block the α-glycosidase enzyme, a critical target for regulating postprandial blood glucose levels, and that this effect was frequently linked to flavonoid concentration in the plant [37]. Research suggests that the plant’s strong saponin content stimulates insulin production from pancreatic β-cells and enhances glucose absorption in peripheral tissues, in addition to blocking carbohydrate-digesting enzymes [5]. Furthermore, a 2024 study found that a mixed aqueous extract of *T. terrestris* and *Curcuma amada* was highly efficient in diabetic rats, drastically decreasing mean blood glucose and glycated hemoglobin (HbA1c) while improving body weight and plasma insulin levels [38]. An 8-week in vivo study found that the aqueous fruit extract of *T. terrestris* was more effective than metformin in restoring normal blood glucose levels and rehabilitating the histological architecture of hepatocytes in diabetic rats, owing to the herb’s potent antioxidant activities, which included direct reactive oxygen species (ROS) scavenging and regeneration of key antioxidant enzymes such as catalase (CAT) and glutathione-S-transferase (GST) [64]. Another study discovered that extracts with high protodioscin content (TT-HPC) were more successful at lowering raised blood glucose levels in diabetic rats than those with low protodioscin concentration (TT-LPC) [5]. This indicates that the concentration of specific saponins plays a crucial role in the plant’s hypoglycemic effect and suggests that *T. terrestris* may synergistically combine with other medicinal plants.

This work presents a fresh and comparative phytochemical and pharmacological profile of *T. terrestris*, focusing on the LE and SE. The significant contribution is the direct evidence that the LE, frequently disregarded in favor of the fruit, exhibits higher bioactivity in numerous domains, including antibacterial, antioxidant, cytotoxic, and antidiabetic assays. The downregulation of Bcl-2 and Bcl-xL genes in cancer cells, combined with the plant’s inhibitory activity against α-glucosidase and α-amylase, provides new insights into its traditional uses and positions it as a promising source for future therapeutic development.

We acknowledge the shortcomings and limitations in terms of the methodology we used in our present study. To ensure consistency in retention times and spectral matches, each GC-MS run for both leaf and seed extracts should be performed in triplicate. To further validate the newly identified compounds, retention indices of the components should be calculated in relation to the retention periods of a series of n-alkanes (two standard mixes, C_8_–C_20_ and C_21_–C_40_) using linear interpolation. It is also recognized that steroidal saponins and other glycosidic chemicals, which are the primary bioactive principles of *T. terrestris*, are non-volatile and thus cannot be detected successfully by GC-MS. Future research will use complementary techniques such as LC-MS/MS and HPLC-ELSD to accomplish a thorough characterization and quantification of steroidal glycosides. These additional tests will offer a more thorough chemical fingerprint of *T. terrestris* and validate the early findings presented here.

## 5. Conclusions

The study demonstrates that *T. terrestris* contains high concentrations of bioactive compounds with significant multifunctional pharmacological potential. The LE consistently proved more potent than the SE, exhibiting vigorous antibacterial activity against Gram-negative pathogens, notable antioxidant capacity, potent cytotoxicity against MCF-7 and HepG2 cancer cells through apoptosis induction, and effective inhibition of key carbohydrate-digesting enzymes. The enhanced efficacy of the LE is closely related to its higher phenolic, flavonoid, and terpenoid content. These results support the ethnopharmacological uses of *T. terrestris* and highlight the LE as a valuable yet underutilized component for future nutraceutical and pharmaceutical research and applications.

## Figures and Tables

**Table 1:** GC-MS compounds in *T. terrestris* LE.

Peak	RT	Area	Area %	Name	MF	MW	Classification
1	9.118	51,513,682	0.97	D-Limonene	C_10_H_16_	136	Monoterpenoids
2	14.547	10,595,389	0.19	2-Decenal, (E)-	C_10_H_18_O	154	Aldehyde
3	16.574	12,551,830	0.23	Linalyl acetate	C_12_H_20_O_2_	196	Monoterpenoids
4	18.824	9,470,910	0.17	3,5-Heptadienal, 2-ethylidene-6-methyl-	C_10_H_14_O	150	Monoterpenoids
5	19.507	2,957,458	0.05	2,6-Dimethyl-8-oxoocta-2,6-dienoic acid, methyl ester	C_11_H_16_O_3_	196	Monoterpenoids
6	21.34	28,681,887	0.54	(E)-Tetradec-2-enal	C_14_H_26_O	210	Fatty aldehydes
7	22.439	9,097,545	0.17	trans-2-undecenoic acid	C_11_H_20_O_2_	184	Fatty acids and conjugates
8	24.031	4,632,250	0.08	[5,5-Dimethyl-6-(3-methyl-buta-1,3-dienyl)-7-oxa-bicyclo [4.1.0]hept-1-yl]-methanol	C_14_H_22_O_2_	222	Oxepanes
9	25.08	7,892,209	0.14	2,4-Di-tert-butylphenol	C_14_H_22_O	206	Phenylpropanes
10	27.515	26,603,932	0.50	1-Hexadecanol, 2-methyl-	C_17_H_36_O	256	Fatty alcohols
11	30.958	12,217,607	0.23	5-O-Methyl-d-gluconic acid dimethylamide	C_9_H_19_NO_6_	237	Fatty amides
12	32.589	28,502,221	0.53	6-O-Methyl-2,4-methylene-β-sedoheptitol	C_9_H_18_O_7_	238	1,3-dioxanes
13	33.088	17,233,900	0.32	Octose	C_8_H_16_O_8_	240	Monosaccharides
14	34.028	11,721,796	0.22	d-Glycero-d-ido-heptose	C_7_H_14_O_7_	210	Monosaccharides
15	34.219	187,227,793	3.52	β-Lactose	C_12_H_22_O_11_	342	Glycosyl compounds
16	34.361	20,427,543	0.38	d-Gala-l-ido-octonic amide	C_8_H_17_NO_8_	255	Monosaccharides
17	34.833	25,284,815	0.47	β-D-Mannofuranoside, 1-O-(10-undecenyl)-	C_17_H_32_O_6_	332	Fatty acyl glycosides of mono- and disaccharides
18	35.292	59,864,528	1.12	Desulphosinigrin	C_10_H_17_NO_6_S	279	Thioglycosides
19	36.914	109,968,411	2.07	Methyl 6-O-[1-methylpropyl]-β-d-galactopyranoside	C_11_H_22_O_6_	250	O-glycosyl compounds
20	37.859	323,542,928	6.09	n-Hexadecanoic acid	C_16_H_32_O_2_	256	Long-chain fatty acids
21	38.159	56,195,332	1.05	Hexadecanoic acid, ethyl ester	C_18_H_36_O_2_	284	Fatty acid esters
22	40.888	343,368,273	6.47	Phytol	C_20_H_40_O	296	Diterpenoid
23	42.02	865,971,414	16.32	Linolenic acid	C_18_H_30_O_2_	278	Linoleic acids and derivatives
24	42.422	38,940,585	0.73	Stearic acid	C_18_H_36_O_2_	284	Long-chain fatty acids
25	43.401	29,301,050	0.55	8,11,14-Eicosatrienoic acid, (Z,Z,Z)-	C_20_H_34_O_2_	306	Long-chain fatty acids
26	44.426	19,150,433	0.36	Arachidonic acid methyl ester	C_21_H_34_O_2_	318	Fatty acid methyl esters
27	44.724	14,142,888	0.26	Methyl 13,16-docosadienoate	C_23_H_42_O_2_	350	Fatty acid methyl esters
28	48.266	32,799,596	0.61	Corynan-17-ol, 18,19-didehydro-10-methoxy-, acetate (ester)	C_22_H_28_N_2_O_3_	368	Indoles and derivatives
29	49.138	14,894,988	0.28	18,19-Secoyohimban-19-oic acid, 16,17,20,21-tetradehydro-16-(hydroxymethyl)-, methyl ester, (15β,16E)-	C_21_H_24_N_2_O_3_	352	Indoles and derivatives
30	49.4	156,996,266	2.95	Palmitic acid β-monoglyceride	C_19_H_38_O_4_	330	Glycerolipids
31	50.135	100,188,763	1.88	26-Nor-5-cholesten-3β-ol-25-one	C_26_H_42_O_2_	386	Oxosteroids
32	51.099	67,851,955	1.27	2-[4-methyl-6-(2,6,6-trimethylcyclohex-1-enyl)hexa-1,3,5-trienyl]cyclohex-1-en-1-carboxaldehyde	C_23_H_32_O	324	Retinoids
33	52.847	715,821,052	13.49	Vitamin E	C_29_H_50_O_2_	430	Vitamin E compounds
34	53.14	371,308,434	6.99	Linolenic acid, methyl ester	C_21_H_36_O_4_	352	Linoleic acids and derivatives
35	53.657	100,137,742	1.88	α-Monostearin	C_21_H_42_O_4_	358	Monoacylglycerols
36	54.116	195,379,212	3.68	Lupeol	C_30_H_50_O	426	Triterpenoid
37	55.159	223,293,715	4.21	Oleamide	C_18_H_35_NO	281	Fatty amides
38	55.426	204,209,299	3.84	Estrone methyl ether	C_23_H_28_O_6_	400	Estrane steroids
39	55.986	77,621,180	1.46	2,2,4-Trimethyl-3-(3,8,12,16-tetramethyl-heptadeca-3,7,11,15-tetraenyl)-cyclohexanol	C_30_H_52_O	428	Triterpenoids
40	56.426	67,809,797	1.27	Cholest-22-ene-21-ol, 3,5-dehydro-6-methoxy-, pivalate	C_33_H_54_O_3_	498	Cholestane steroids
41	57.077	396,141,007	7.46	Campesterol	C_28_H_48_O	400	Ergosterols and derivatives
42	58.971	254,003,805	4.78	Stigmasterol	C_29_H_48_O	412	Stigmastanes and derivatives

Note: Retention time (RT), molecular formula (MF), and molecular weight (MW).

**Table 2:** GC-MS compounds in *T. terrestris* SE.

Peak	RT	Area	Area%	Name	MF	MW	Classification
1	9.026	27,674,175	1.18	D-Limonene	C_10_H_16_	136	Monoterpenoids
2	12.583	1,292,485	0.05	1-Azabicyclo [3.2.1]octan-6-ol,	C_7_H_13_NO	127	Azepanes
3	14.539	6,362,755	0.27	2-Decenal, (E)-	C_10_H_18_O	154	Aldehyde
4	16.283	27,132,745	1.16	2-Ethyl-2-hexen-1-ol	C_8_H_16_O	128	Fatty alcohols
5	16.578	4,377,573	0.18	2,2-Dimethyl-5-(3-methyl-2-oxiranyl)cyclohexanone	C_11_H_18_O_2_	182	Cyclic ketones
6	17.073	27,542,799	1.17	Diplodialide B	C_10_H_16_O_3_	184	Oxocins
7	17.39	8,535,962	0.36	R-Limonene	C_10_H_16_O_3_	184	Monoterpenoids
8	18.416	18,070,103	0.77	9-Tetradecenal, (Z)-	C_14_H_26_O	210	Fatty aldehydes
9	20.567	119,036,249	5.09	13-Tetradece-11-yn-1-ol	C_14_H_24_O	208	Fatty alcohols
10	21.341	64,046,996	2.74	(E)-Tetradec-2-enal	C_14_H_26_O	210	Fatty aldehydes
11	21.702	27,011,428	1.15	3-Nonynoic acid	C_9_H_14_O2	154	Fatty acids and conjugates
12	22.239	21,603,631	0.92	cis,cis-7,10,-Hexadecadienal	C_16_H_28_O	236	Fatty aldehydes
13	24.353	11,191,061	0.47	11-Hexadecyn-1-ol	C_16_H_30_O	238	Fatty alcohol
14	24.564	9,064,484	0.38	13-Heptadecyn-1-ol	C_17_H_32_O	252	Fatty alcohol
15	24.83	14,909,604	0.63	E-11-Hexadecenal	C_16_H_30_O	238	Fatty aldehydes
16	27.09	10,712,686	0.45	7-Methyl-Z-tetradecen-1-ol acetate	C_17_H_32_O_2_	268	Fatty alcohol esters
17	27.498	25,117,256	1.07	1-Hexadecanol, 2-methyl-	C_17_H_36_O	256	Fatty alcohol
18	27.845	6,916,745	0.29	9-Hexadecenoic acid	C_16_H_30_O_2_	254	Fatty acids and conjugates
19	32.54	22,489,431	0.96	6-O-Methyl-2,4-methylene-β-sedoheptitol	C_9_H_18_O_7_	238	Dioxanes
20	33.069	17,832,901	0.76	Octose	C_8_H_16_O_8_	240	Monosaccharides
21	34.177	12,983,214	0.55	β-Lactose	C_12_H_22_O_11_	342	Glycosyl compounds
22	34.329	5,968,022	0.25	d-Gala-l-ido-octonic amide	C_8_H_17_NO_8_	255	Monosaccharides
23	35.844	18,000,733	0.77	Desulphosinigrin	C_10_H_17_NO_6_S	279	Thioglycosides
24	37.787	266,140,032	11.38	n-Hexadecanoic acid	C_16_H_32_O_2_	256	Long-chain fatty acids
25	38.135	61,588,053	2.63	Hexadecanoic acid, ethyl ester	C_18_H_36_O_2_	284	Fatty acid esters
26	40.816	25,044,348	1.07	2-(Octadec-9-enyloxy)ethanol	C_20_H_40_O_2_	312	Ethers
27	41.745	281,852,469	12.05	cis,cis-Linoleic acid	C_18_H_32_O_2_	280	Linoleic acids and derivatives
28	41.848	179,843,343	7.69	Linolenic acid	C_18_H_30_O_2_	278	Linoleic acids and derivatives
29	41.878	124,694,063	5.33	Linoleic acid ethyl ester	C_20_H_36_O_2_	308	Linoleic acids and derivatives
30	42.104	24,879,985	1.06	Ethyl Oleate	C_20_H_38_O_2_	310	Fatty acid esters
31	42.331	62,232,484	2.66	Oleic Acid	C_18_H_34_O_2_	282	Fatty acids and conjugates
32	42.749	15,226,202	0.65	(E)-9-Octadecenoic acid ethyl ester	C_20_H_38_O_2_	310	Fatty acid esters
33	43.077	22,693,098	0.97	Linolenic acid, methyl ester	C_21_H_36_O_4_	352	Linoleic acids and derivatives
34	44.39	13,034,909	0.55	Arachidonic acid methyl ester	C_21_H_34_O_2_	318	Fatty acid methyl esters
35	44.683	9,712,486	0.41	Methyl 13,16-docosadienoate	C_23_H_42_O_2_	350	Fatty acid methyl esters
36	44.903	6,506,007	0.27	Glyceryl diacetate 2-linolenate	C_25_H_40_O_6_	436	Triacylglycerols
37	45.047	5,778,806	0.24	Ethyl iso-allocholate	C_26_H_44_O_5_	436	Hydroxy bile acids, alcohols and derivatives
38	48.103	16,807,940	0.71	Cholestan-3-ol, 2-methylene-, (3β,5α)-	C_28_H_48_O	400	Cholesterols and derivatives
39	48.235	5,805,416	0.24	Corynan-17-ol, 18,19-didehydro-10-methoxy-, acetate (ester)	C_22_H_28_N_2_O_3_	368	Indoles and derivatives
40	48.395	18,970,108	0.81	2-Monolinolenin	C_21_H_36_O_4_	352	Linoleic acids and derivatives
41	48.523	8,012,896	0.34	2-Monoolein	C_21_H_40_O_4_	356	Glycerolipids
42	49.109	18,191,454	0.77	18,19-Secoyohimban-19-oic acid, 16,17,20,21-tetradehydro-16-(hydroxymethyl)-, methyl ester, (15β,16E)-	C_21_H_24_N_2_O_3_	352	Indoles and derivatives
43	49.363	106,419,555	4.55	Palmitic acid β-monoglyceride	C_19_H_38_O_4_	330	Glycerolipids
44	52.87	202,940,007	8.68	1-Monolinolein	C_21_H_38_O_4_	354	monoacylglycerol
45	53.596	48,811,429	2.08	α-Monostearin	C_21_H_42_O_4_	358	Monoacylglycerols
46	55.134	304,089,901	13.01	Oleamide	C_18_H_35_NO	281	Fatty amides

Note: Retention time (RT), molecular formula (MF), and molecular weight (MW).

**Table 3:** The inhibitory zone (mm), MIC (μg/mL), and MBC (μg/mL) of *T. terrestris* LE.

Bacterium/ Dilution	Positive Control	1000 μg/mL	500 μg/mL	250 μg/mL	125 μg/mL	MIC (μg/mL)	MBC (μg/mL)
*S. aureus*	29 ± 0.00	24 ± 0.00 *	20 ± 0.00 *	16 ± 0.00 *	11 ± 0.00 *	25 ± 0.00	50 ± 0.00
*S. epidermidis*	31 ± 0.00	25 ± 0.00 *	21 ± 0.00 *	17 ± 0.00 *	12 ± 0.00 *	25 ± 0.00	50 ± 0.00
*B. subtilis*	27 ± 0.00	23 ± 0.00 *	19 ± 0.00 *	14 ± 0.00 *	11 ± 0.00 *	25 ± 0.00	50 ± 0.00
*E. coli*	30 ± 0.00	25 ± 0.00 *	22 ± 0.00 *	17 ± 0.00 *	14 ± 0.00 *	12.50 ± 0.00	25 ± 0.00
*K. pneumoniae*	22 ± 0.00	17 ± 0.00 *	14 ± 0.00 *	10 ± 0.00 *	8 ± 10.00	6.25 ± 0.00	12.50 ± 0.00
*P. aeruginosa*	26 ± 0.00	24 ± 0.00 *	21 ± 0.00 *	14 ± 0.00 *	11 ± 0.00 *	12.50 ± 0.00	25 ± 0.00

Note: minimum inhibitory concentration (MIC), minimum bactericidal concentration (MBC). The reported values are shown in triplicate as mean ± SD. The results demonstrate a statistically significant decrease from the positive control (25 µg/mL of chloramphenicol), indicated by (* *p* < 0.01).

**Table 4:** The inhibitory zone (mm), MIC (μg/mL), and MBC (μg/mL) of *T. terrestris* SE.

Bacterium/ Dilution	Positive Control	1000 μg/mL	500 μg/mL	250 μg/mL	125 μg/mL	MIC (μg/mL)	MBC (μg/mL)
*S. aureus*	29 ± 0.00	22 ± 0.00 *	17 ± 0.00 *	15 ± 0.00 *	14 ± 0.00 *	25 ± 0.00	50.00 ± 0.00
*S. epidermidis*	31 ± 0.00	23 ± 0.00 *	19 ± 0.00 *	16 ± 0.00 *	13 ± 0.00 *	25 ± 0.00	50 ± 0.00
*B. subtilis*	27 ± 0.00	20 ± 0.00 *	18 ± 0.00 *	15 ± 0.00 *	10 ± 0.00 *	50 ± 0.00	100 ± 0.00
*E. coli*	30 ± 0.00	24 ± 0.00 *	18 ± 0.00 *	12 ± 0.00 *	10 ± 0.00 *	12.50 ± 0.00	50.00 ± 0.00
*K. pneumoniae*	22 ± 0.00	20 ± 0.00 *	16 ± 0.00 *	12 ± 0.00 *	9 ± 10.00 *	12.50 ± 0.00	50 ± 0.00
*P. aeruginosa*	26 ± 0.00	26 ± 0.00	20 ± 0.00 *	17 ± 0.00 *	13 ± 0.00 *	25 ± 0.00	50 ± 0.00

Note: minimum inhibitory concentration (MIC), minimum bactericidal concentration (MBC). The reported values are shown in triplicate as mean ± SD. The results demonstrate a statistically significant decrease from the positive control (25 µg/mL of chloramphenicol), indicated by (* *p* < 0.01).

## Data Availability

Data is contained within the article.
